# Dihydrotestosterone potentiates insulin to up‐regulate prokineticin‐1 in decidualizing human endometrial stromal cells

**DOI:** 10.1111/jcmm.14923

**Published:** 2020-01-28

**Authors:** Dorina Ujvari, Carlota Graells Brugalla, Angelica Lindén Hirschberg

**Affiliations:** ^1^ Department of Women’s and Children’s Health Karolinska Institutet Stockholm Sweden; ^2^ Department of Gynecology and Reproductive Medicine Karolinska University Hospital Stockholm Sweden

**Keywords:** decidualization, dihydrotestosterone, endometrium, insulin, PROK1, testosterone

## Abstract

Prokineticin 1 (PROK1) is a key regulator of embryo implantation and placentation, and its dysregulation is associated with pregnancy complications, such as pre‐eclampsia and foetal growth restriction. We have previously shown that insulin strongly enhances the expression of PROK1 in human decidualizing stromal cells. Here, we demonstrate that dihydrotestosterone (DHT), but not testosterone, potentiates insulin to up‐regulate PROK1 in these cells. However, the androgens alone do not influence the expression of PROK1. Our findings suggest that insulin and androgens both are involved in the regulation of PROK1 that could have implications for normal and pathological pregnancies.

## 1 INTRODUCTION

Trophoblast invasion of decidua and maternal spiral arteries is the key process in the establishment of feto‐maternal circulation in early pregnancy. Prokineticin 1 (PROK1) is highly expressed in female reproductive organs, such as ovaries, placenta and endometrium, and has emerged as a crucial negative regulator of extravillous trophoblast invasion.^1^, ^2^ PROK1 is predominantly regulated by oxygen tension and its expression dynamically changes during pregnancy. Maternal serum levels are relatively high in the first trimester, but falls after pregnancy week 10‐11, suggesting an essential role for PROK1 in early pregnancy.^3^ In early pregnancy, PROK1 promotes the proliferation of cytotrophoblasts at the expense of their differentiation and by this contributes to the formation and maintenance of trophoblastic plugs in the maternal spiral arteries that protects the developing foetus and placenta from highly oxygenated maternal blood. At week 10‐11, the decrease in circulating and placental levels of PROK1 possibly contributes to differentiation and invasion of extravillous trophoblasts into the decidua and remodelling the maternal spiral arteries to ensure sufficient amount of oxygen and nutrients for the growing foetus.^2^ Dysregulation of PROK1 has been associated with placenta‐related pregnancy complications, such as pre‐eclampsia and intrauterine growth restriction.^2^, ^3^, ^4^

Recently, we showed that insulin highly up‐regulates PROK1 in decidualizing human stromal cells.^5^ Hypothetically, this could be of clinical relevance, since insulin resistance and compensatory hyperinsulinemia are associated with increased risk of placenta‐related pregnancy complications.^6^, ^7^ Furthermore, indirect evidence supports the role of androgens in the pathogenesis of placenta‐related pregnancy complications as shown in polycystic ovary syndrome (PCOS), a metabolic disorder, which is associated with both hyperinsulinemia and hyperandrogenism.^8^, ^9^, ^10^ These findings prompted us to explore the effect of androgens on the PROK1 expression in decidualizing stromal cells alone and in combination with insulin.

## 2 METHODS

Endometrial biopsies were collected from nine regularly cycling, healthy volunteers between age 18‐35 and body mass index 19‐28 on cycle day 5‐9 as previously described.^11^ Exclusion criteria were described elsewhere.^11^ The Regional Ethical Committee in Stockholm approved the study (Dnr 2018/2199‐31). A written consent was obtained from all participants.

Isolation of endometrial stromal cells and in vitro decidualization using 0.5 mmol/L N^6^, 2'‐O‐dibutyryladenosine cAMP and 1 μmol/L medroxyprogesterone‐17‐acetate were carried out as previously described.^11^ Cells were co‐treated with 100 nmol/L insulin (Sigma‐Aldrich, USA), 1 μmol/L DHT (Sigma‐Aldrich, USA) or 1 μmol/L testosterone (Sigma‐Aldrich, USA), and the combination of insulin+DHT or insulin+testosterone. All treatments were carried out under normoxic conditions and were performed at least twice. Culture media and treatments were renewed after 3 days. On day 6 conditioned media were collected, and cells were harvested for RNA extraction.

Total RNA was extracted, and cDNA was synthesized as described previously.^5^ 10 ng cDNA was subjected to RT‐PCR to measure PROK1 and housekeeping gene, ribosomal protein L13a mRNA using TaqMan assays Hs00168730_m1 and Hs01926559_g1 (Thermo Fischer Scientific, USA), respectively. The reactions were run in triplicate, and gene expression levels were determined using the ΔΔC_T_ method.

Secreted PROK1 protein was measured from undiluted conditioned media using ELISA (PeproTech, USA) according to the manufacturer's instruction. The limit of detection (LOD) was 16 pg/mL. Values below the LOD were replaced by half of LOD.

All data were log‐transformed, and normal distribution was challenged with the Kolmogorov–Smirnov test. Two‐way ANOVA with Sidak's multiple comparison test was used to determine the effect of insulin and/or androgen exposure.

## 3 RESULTS

**Figure 1:**
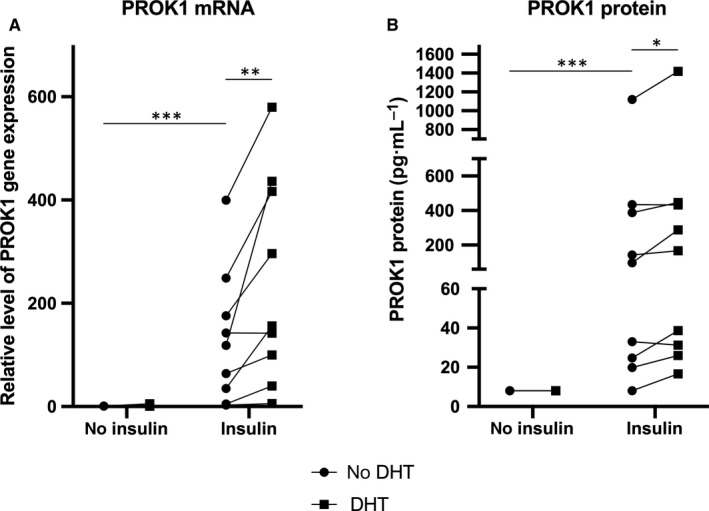
A, Relative level of PROK1 gene expression in nine healthy volunteers in response to 100 nmol/L insulin and/or 1 µmol/L DHT in in vitro decidualized human endometrial stromal cells after 6 d. Closed circles represent treatments without DHT, closed squares represent treatments with DHT. Main effect of insulin: *P* < .001; main effect of DHT: ns; interaction effect between insulin and DHT: *P* < .05. Sidak`s multiple comparison test: **P < .01; ***P < .001. B, PROK1 protein expression in nine healthy volunteers in response to 100 nmol/L insulin and/or 1 µmol/L DHT in in vitro decidualized human endometrial stromal cells after 6 days. Closed circles represent treatments without DHT, closed squares represent treatments with DHT. Main effect of insulin: *P* < .01; main effect of DHT: ns; interaction effect between insulin and DHT: *P* < .05. Sidak`s multiple comparison test: *P < .05; ***P < .001

**Figure 2:**
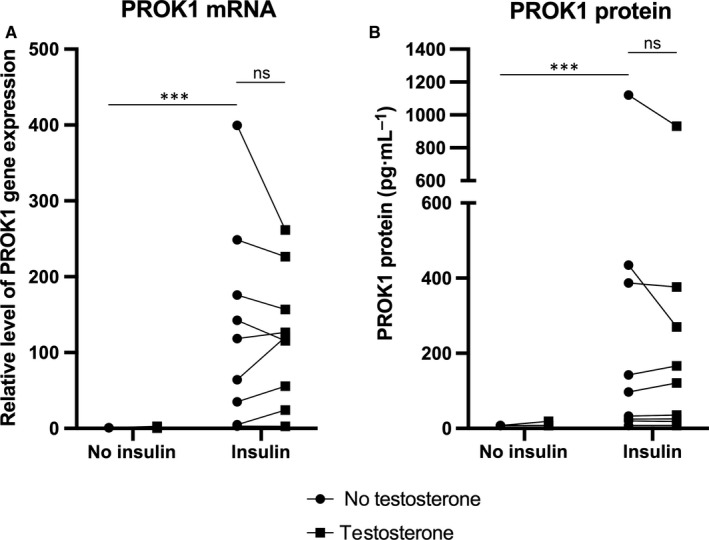
A, Relative level of PROK1 gene expression in nine healthy volunteers in response to 100 nmol/L insulin and/or 1 µmol/L testosterone in in vitro decidualized human endometrial stromal cells after 6 d. Closed circles represent treatments without testosterone, closed squares represent treatments with testosterone. Main effect of insulin: *P* < .001; main effect of testosterone: ns; interaction effect between insulin and testosterone: ns. Sidak's multiple comparison test: ***P < .001. B, PROK1 protein expression in nine healthy volunteers in response to 100 nmol/L insulin and/or 1 µmol/L testosterone in in vitro decidualized human endometrial stromal cells after 6 days. Closed circles represent treatments without testosterone, closed squares represent treatments with testosterone. Main effect of insulin: *P* < .01; main effect of testosterone: ns; interaction effect between insulin and testosterone: ns. Sidak's multiple comparison test: ***P < .001

There was a main effect of insulin exposure in decidualized stromal cells with increased PROK1 at mRNA and protein level. We could not detect main effect of DHT/testosterone exposure. There was an interaction effect between insulin and DHT at both mRNA and protein level, but no interaction between insulin and testosterone. Insulin enhanced both gene (*P* < .001) and protein expression of PROK1 (*P* < .001) in human decidualized stromal cells, while neither DHT nor testosterone had a significant effect alone on PROK1 expression when compared to treatment with decidualization agents (MPA and db‐cAMP) alone. Insulin+DHT or insulin+testosterone also significantly increased both gene and protein expression of PROK1 compared with treatment with only decidualization agents (*P* < .001 and *P* < .001, respectively). Insulin+DHT or insulin+testosterone significantly increased PROK1 gene (*P* < .001 and *P* < .001, respectively) and protein expression (*P* < .001 and *P* < .001, respectively) compared with DHT or testosterone treatment. Insulin + DHT enhanced both gene (*P* < .01) and protein expression of PROK1 (*P* < .05) even more in comparison with insulin treatment (Figures 1 and 2).

## 4 DISCUSSION

Here, we demonstrated that DHT or testosterone exposure alone has no effect on PROK1 in decidualizing stromal cells, however, DHT, but not testosterone, potentiates insulin to increase PROK1 at mRNA and protein level. The difference between the effect of testosterone and DHT might be partially explained by the twofold higher affinity and fivefold decreased dissociation rate of DHT to the androgen receptor compared with testosterone.^12^ Furthermore, testosterone can be converted to estradiol by aromatase, while DHT is non‐aromatizable. As the presence of functional aromatase was evaluated by the increasing production of estrone and estradiol in decidualizing endometrial stromal cells,^13^ further studies need to elucidate a potential effect of these hormones on the regulation of PROK1. We recently demonstrated that hypoxia‐inducible factor‐1α is involved in the up‐regulation of PROK1 by insulin.^5^ However, the mechanism of the combined action of insulin and androgens on PROK1 needs to be further explored.

Experimental evidence suggests that PROK1 promotes proliferation, but impairs migration and invasion of trophoblast cells and their differentiation in order to remodel the maternal spiral arteries.^3^, ^5^, ^14^ Several publications propose the involvement of androgens in the pathogenesis of placenta‐related pregnancy complications. It was shown that trophoblast invasion and placentation are impaired in women with PCOS and endovascular trophoblast invasion is indirectly related to circulating testosterone concentration.^8^ Moreover, increased maternal serum androgens have been well‐characterized in pre‐eclamptic pregnancies,^9^, ^10^ and women with PCOS and pregnancy complications showed higher androgen levels than those without.^15^

In conclusion, our results suggest that insulin and androgens both are involved in the regulation of PROK1 in human decidualizing stromal cells. This could be of relevance for implantation, placentation and the development of placenta‐related pregnancy complications.

## CONFLICTS OF INTEREST

The authors declare no conflicts of interest.

## AUTHOR CONTRIBUTION

DU and ALH designed the study; ALH enrolled the participants and took the endometrial biopsies; DU and CGB performed the experiments; DU and CGB analysed and interpreted the data; DU visualized the data; DU wrote the initial draft of the manuscript; ALH had overall responsibility for the study; all authors contributed to writing the manuscript, have seen, reviewed and approved the final version of the manuscript.

## Data Availability

The data that support the findings of this study are available from the corresponding author upon reasonable request.
